# *Tectus niloticus* (Tegulidae, Gastropod) as a Novel Vector of Ciguatera Poisoning: Detection of Pacific Ciguatoxins in Toxic Samples from Nuku Hiva Island (French Polynesia)

**DOI:** 10.3390/toxins10010002

**Published:** 2017-12-21

**Authors:** Hélène Taiana Darius, Mélanie Roué, Manoella Sibat, Jérôme Viallon, Clémence Mahana iti Gatti, Mark W. Vandersea, Patricia A. Tester, R. Wayne Litaker, Zouher Amzil, Philipp Hess, Mireille Chinain

**Affiliations:** 1 Institut Louis Malardé (ILM), Laboratory of Toxic Microalgae—UMR 241-EIO, P.O. Box 30, 98713 Papeete, Tahiti, French Polynesia; jviallon@ilm.pf (J.V.); cgatti@ilm.pf (C.M.i.G.); mchinain@ilm.pf (M.C.); 2 Institut de Recherche pour le Développement (IRD)—UMR 241-EIO, P.O. Box 529, 98713 Papeete, Tahiti, French Polynesia; melanie.roue@ird.fr; 3 IFREMER, Phycotoxins Laboratory, F-44311 Nantes, France; manoella.sibat@ifremer.fr (M.S.); zouher.amzil@ifremer.fr (Z.A.); philipp.hess@ifremer.fr (P.H.); 4 National Oceanic and Atmospheric Administration, National Ocean Service, Centers for Coastal Ocean Science, Beaufort Laboratory, Beaufort, NC 28516, USA; mark.w.vandersea@noaa.gov (M.W.V.); wayne.litaker@noaa.gov (R.W.L.); 5 Ocean Tester, LLC, Beaufort, NC 28516, USA; ocean.tester@gmail.com

**Keywords:** ciguatera poisoning, ciguatoxins, *Tectus niloticus*, gastropods, *Gambierdiscus polynesiensis*, qPCR assays, CBA-N2a, LC-MS/MS

## Abstract

Ciguatera fish poisoning (CFP) is a foodborne disease caused by the consumption of seafood (fish and marine invertebrates) contaminated with ciguatoxins (CTXs) produced by dinoflagellates in the genus *Gambierdiscus*. The report of a CFP-like mass-poisoning outbreak following the consumption of *Tectus niloticus* (Tegulidae, Gastropod) from Anaho Bay on Nuku Hiva Island (Marquesas archipelago, French Polynesia) prompted field investigations to assess the presence of CTXs in *T. niloticus*. Samples were collected from Anaho Bay, 1, 6 and 28 months after this poisoning outbreak, as well as in Taiohae and Taipivai bays. Toxicity analysis using the neuroblastoma cell-based assay (CBA-N2a) detected the presence of CTXs only in Anaho Bay *T. niloticus* samples. This is consistent with qPCR results on window screen samples indicating the presence of *Gambierdiscus* communities dominated by the species *G. polynesiensis* in Anaho Bay. Liquid chromatography-tandem mass spectrometry (LC-MS/MS) analyses revealed that P-CTX-3B was the major congener, followed by P-CTX-3C, P-CTX-4A and P-CTX-4B in toxic samples. Between July 2014 and November 2016, toxin content in *T. niloticus* progressively decreased, but was consistently above the safety limit recommended for human consumption. This study confirms for the first time *T. niloticus* as a novel vector of CFP in French Polynesia.

## 1. Introduction

*Tectus niloticus* Linnaeus, 1767 (Gastropoda, Trochoidea, Tegulidae), a large, herbivorous marine snail widespread throughout Southeast Asia and the Western Pacific, is commonly known as trochus or top shell [1,2,3]. It can be found in both intertidal and subtidal reef habitats, generally in high energy sections of the reef. The optimum depth for *T. niloticus* is between 0 and 15 m, although individuals can be found as deep as 25 m [3,4]. Juveniles are quite cryptic, but larger individuals are often found on tropical coral reef flats [3,5]. *T. niloticus* are herbivores and tend to eat turf algae and biofilm by grazing on corals and rocks [6,7].

*T. niloticus* has been introduced to many South Pacific Islands and is usually fished artisanally for its shell, which is used to make traditional ornaments by indigenous people, as well as in the manufacture of mother-of-pearl buttons, which can be a valuable source of income for people in the Indo-Pacific region [2,6,8,9]. The shell is also used in cosmetics and paint [3,10]. In addition, *T. niloticus* meat represents a valuable source of protein for many communities in Asian, as well as Pacific Island Countries and Territories (PICTs) [6,11]. Primary harvesters of trochus shells include Indonesia, the Philippines and Thailand, while Japan, Hong Kong and Europe are the most important consumers [10,12]. 

*T. niloticus* was introduced in French Polynesia from the Republic of Vanuatu as early as 1957 to establish a fishery [13], but due to abusive exploitation, a law was issued in 1988 to protect this resource through a permanent fishing closure. However, there are limited fishing seasons authorized by the government with size limits and set quotas for the trade of mother-of-pearl and for consumption by local populations [14].

In June 2014, a mass-poisoning outbreak involving nine tourists occurred in Nuku Hiva Island (Marquesas archipelago, French Polynesia) following the consumption of *T. niloticus* collected from Anaho Bay [15,16]. All patients exhibited clinical symptoms typical of ciguatera fish poisoning (CFP), i.e., gastrointestinal (vomiting/diarrhea), cardiovascular (bradycardia and hypotension) and neurological (asthenia/myalgia, paresthesias/dysesthesias/cold allodynia) disorders, as well as symptoms like burning/tingling sensation of the mouth and throat [15,16]. Of note were atypical features such as the rapid onset (2 h) and unusual severity of gastro-intestinal and neurological symptoms, which necessitated the hospitalization of six patients [15,16]. 

CFP is a food-borne poisoning known to be caused primarily by the ingestion of coral reef fishes contaminated by potent neurotoxins, ciguatoxins (CTXs), originating from benthic dinoflagellates in the genus *Gambierdiscus* [17,18]. However, ciguatera poisoning incidents involving marine invertebrates such as giant clams (e.g., *Tridacna maxima*, *Hippopus hippopus*) or sea urchins (*Tripneustes gratilla*) are also currently reported in PICTs (French Polynesia, New Caledonia, Cook Islands, the Republic of Vanuatu, etc.) [19,20,21,22]. A recent study by Roué et al. (2016) provided confirmation that giant-clams (*T. maxima*) are actually able to bioaccumulate CTXs when exposed to *Gambierdiscus* cells [23].

Since *T. niloticus* are herbivores and commonly graze on epiphytic organisms or those growing on biofilms [7], they can potentially bioaccumulate marine biotoxins in their tissues, e.g., upon feeding on toxic micro-algae from benthic assemblages. The aim of this study was to assess the toxicity of *T. niloticus* specimens collected from the toxic area of Anaho Bay over a two-year period and compare the data with two other sites on Nuku Hiva Island, Taipivai and Taiohae Bays. In addition, quantitative polymerase chain reaction (qPCR) assays were used to identify dominant *Gambierdiscus* species present at the sampling sites, as well as in clonal cultures established from the field-collected material. Toxicological analyses were carried out using the neuroblastoma cell-based assay (CBA-N2a) to test for the potential presence of toxins in *T. niloticus* samples. In addition, liquid chromatography coupled to tandem mass spectrometry (LC-MS/MS) was used for both identification of ciguatoxin analogues and multi-toxin screening for a variety of other marine toxins. These include: neurologic shellfish toxins (NSP), i.e., brevetoxins (PbTX1 to PbTX10), paralytic shellfish poisoning (PSP) toxins, i.e., carbamates (STX, NEO-STX, GTX1-GTX4), *N*-sulfocarbamoyl (GTX5, GTX6 and C1 toC4) and decarbamoyls (dcSTX, dcNEO, dcGTX1-dcGTX4); diarrhetic shellfish poisoning (DSP) toxins i.e., okadaic acid (OA), dinophysistoxins (DTXs), pectenotoxins (PTXs), azaspiracids (AZAs) and yessotoxins (YTXs); cyclic imines (fast action toxins (FAT)): gymnodimines (GYMs), spirolides (SPXs) and pinnatoxins (PnTXs).

## 2. Results

### 2.1. Abundance of Gambierdiscus spp. in Study Sites

*Gambierdiscus* cells were collected from macroalgae, as well as from 18 window screen (WS) sampling devices deployed at three sites on Nuku Hiva Island during a field mission in November 2016 (Table 1 and Table 2).

Manual cell counts revealed that macroalgal substrates yielded very low cell densities (i.e., 1.5 ± 2.6 cells/g of macroalgae) with cells being observed only in turf and *Halimeda* samples from Anaho Bay (Table 1). No *Gambierdiscus* cells were counted in Taipivai Bay, while no macroalgae have been found in Taiohae Bay.

Conversely, *Gambierdiscus* cells were collected from every sampling site using WS sampling methods. Ten semi-quantitative, species-specific qPCR assays were used to survey the WS samples for relative cell abundance and *Gambierdiscus* species distribution. Five species were detected including *Gambierdiscus caribaeus*, *G. carpenteri*, *G. pacificus*, *G. polynesiensis* and *G. toxicus* (Table 2). In contrast, *Gambierdiscus australes*, *G. belizeanus*, *G. carolinianus*, *Fukuyoa ruetzleri*, *G. silvae* and *Gambierdiscus* ribotype II were not detected in the WS samples.

*G. polynesiensis* was the predominant species detected in Anaho Bay, whereas *G. carpenteri* predominated in Taiohae and Taipivai Bays (Table 2). *G. toxicus*, *G. caribaeus* and *G. pacificus* were detected at trace concentrations in the WS samples whatever the site (Table 2). 

In parallel, several *Gambierdiscus* clonal cultures were also established from cells collected on window screens. Among the 17 cultured strains originating from Nuku Hiva, *G. carpenteri* was the most prevalent species since 13 strains belonged to *G. carpenteri*, 3 strains belonged to *G. pacificus* and only 1 strain belonged to *G. polynesiensis* (Table 3). None of these strains belonged to *G. caribaeus* or *G. toxicus*. Surprisingly, the *G. polynesiensis* strain was established successfully from material originating from Taiohae Bay where it was present at low densities on WS (10%) (Table 2). In the same way, three strains of *G. pacificus* were established from samples collected from Taiohae and Taipivai Bays where *G. pacificus* was also detected at low densities (<1 cell/150 cm^−2^). Conversely, *G. polynesiensis* was not successfully isolated in culture from Anaho Bay WS samples, although it was the most prevalent species in this area (82%) (Table 2). These observations indicate that the culturing approach may give a highly biased representation of the community species composition.

### 2.2. Toxicity Results Using CBA-N2a

#### 2.2.1. Calibration of CBA-N2a

**Figure 1:**
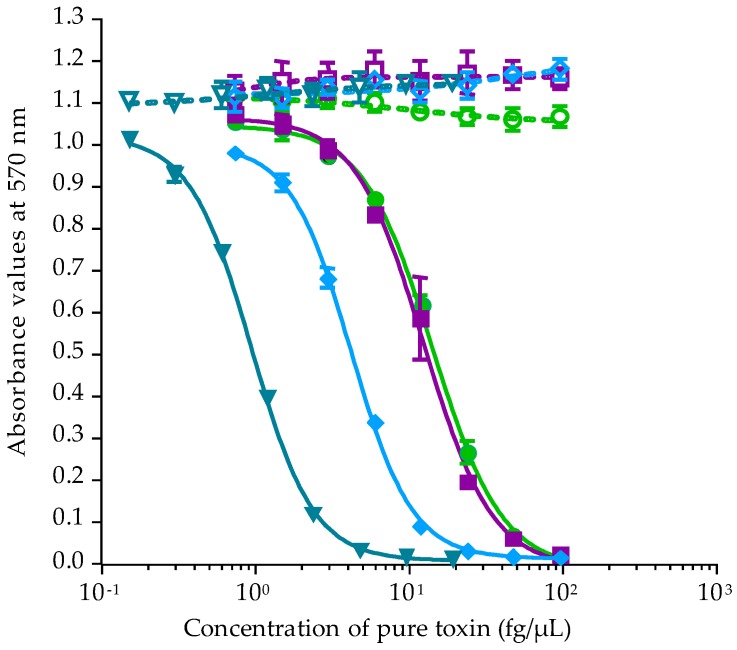
Dose-response curves of Neuro-2a cells in OV^−^ (open symbols) and OV^+^ (solid symbols) conditions, when exposed to increasing concentrations of P-CTX-3C (**▽**/**▼**), P-CTX-3B (**◇**/◆), P-CTX-4A (□/■) and P-CTX-4B (**○**/●) following the CBA-N2a procedure described in Section 4.4. Data represent the mean ± SD of one experiment (each concentration run in triplicate).

Calibration of CBA-N2a was performed using P-CTX-3C, P-CTX-3B, P-CTX-4A and P-CTX-4B standards obtained from the Louis Malardé Institute’s bank of standards. Under OV^+^ conditions (cells treated with ouabain and veratridine mixture), neuro-2a cells typically displayed a sigmoid dose-response curve in the presence of P-CTXs, whereas no cytotoxicity was observed under OV^−^ conditions (untreated cells without ouabain and veratridine added) (Figure 1). Concentrations effective at 50% (EC_50_ values) obtained for P-CTX-3C, P-CTX-3B, P-CTX-4A and P-CTX-4B were 1.44 ± 0.70 (*n* = 9), 5.20 ± 1.34 (*n* = 7), 12.95 ± 1.89 (*n* = 8) and 12.08 ± 4.13 (*n* = 6) fg/µL, respectively. The most potent toxin was P-CTX-3C, followed by P-CTX-3B, with P-CTX-4A and P-CTX-4B showing similar potency (Figure 1). The CBA-N2a was also calibrated with P-CTX-1B (EC_50_ = 1.94 ± 0.41 fg/µL, *n* = 5) for further comparison of the toxin content in *T. niloticus* with both the U.S. Food and Drug Administration (US-FDA) and the European Food Safety Authority (EFSA) advisory levels (0.01 ng equiv./g of flesh) [24,25].

#### 2.2.2. Toxicity of *Gambierdiscus* In Vitro Cultures

**Figure 2:**
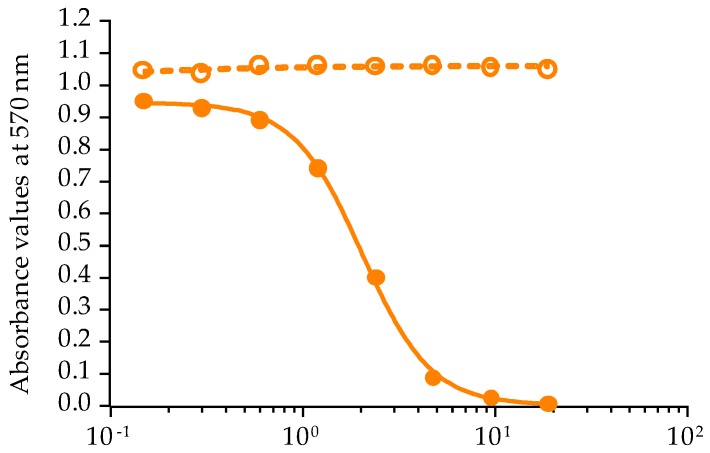
Dose-response curves of neuro-2a cells in OV^−^ (**○**) and OV^+^ (●) conditions, when exposed to increasing concentrations of liposoluble fraction of *G. polynesiensis* strain TIO-10 isolated from Taiohae Bay. Data represent the mean ± SD of one experiment (each concentration run in triplicate).

Among the 17 in vitro cultures tested by CBA-N2a, only *G. polynesiensis* strain TIO-10 originating from Taiohae was found to produce P-CTXs, as shown in Figure 2. Indeed, its liposoluble fraction showed no cytotoxicity under OV^−^ conditions, whereas a sigmoidal dose-response curve was observed in OV^+^ conditions, a response typical of CTXs bioactivity. Its raw EC_50_ was 1.92 ± 0.22 pg/µL of dry extract (*n* = 2) corresponding to (1.21 ± 0.14) × 10^−3^ cell equiv./µL. The mean toxin content in *G. polynesiensis* TIO-10 was thus estimated to be 1.20 ± 0.14 pg P-CTX-3C equiv./cell.

#### 2.2.3. Toxicity of *T. niloticus* Samples

The toxicity of *T. niloticus* extracts obtained from field samples collected from the three study sites was investigated by examining both liposoluble fractions (LF70/30, LF90/10, LF100) and hydrosoluble fractions (HF50/50, HF70/30, HF90/10, HF100). 

1. Samples from Anaho Bay

**Figure 3:**
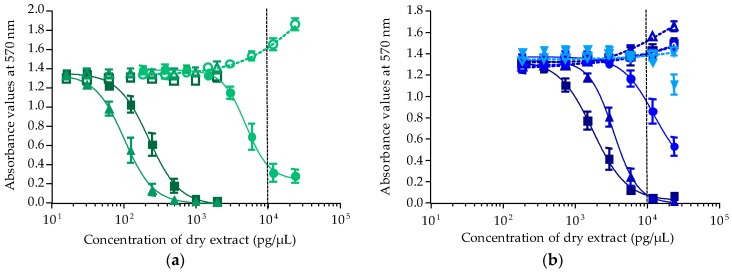
Dose-response curves of neuro-2a cells in OV^−^ (open symbols) and OV^+^ (solid symbols) conditions, when exposed to increasing concentrations of *T. niloticus* fractions (Anaho Bay July 2014). (**a**) Liposoluble fractions LF70/30 (**○**/●), LF90/10 (**△**/**▲**) and LF100 (**□/**■); (**b**) hydrosoluble fractions HF50/50 (**▽**/**▼**), HF70/30 (**○**/●), HF90/10 (**△**/**▲**) and HF100 (**□**/■). Data represent the mean ± SD of two independent experiments (each run in triplicate). The dotted vertical line corresponds to the maximum concentration of dry extract (MCE = 10,000 pg/µL) for matrix interferences.

Except for HF50/50, all *T. niloticus* fractions corresponding to samples collected from Anaho Bay in July 2014 displayed a negative response in OV^−^ conditions and a typical sigmoidal dose-response curve in OV^+^ conditions, a result typical of the mode of action of CTXs (Figure 3a,b). 

**Figure 4:**
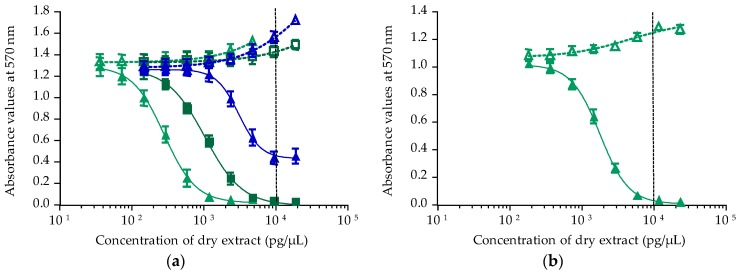
Dose-response curves of neuro-2a cells in OV^−^ (open symbols) and OV^+^ (solid symbols) conditions, when exposed to increasing concentrations of *T. niloticus* extracts from Anaho Bay. (**a**) Samples collected in December 2014; (**b**) samples collected in November 2016. Liposoluble fractions are represented as follows: LF90/10 (**△**/**▲**) and LF100 (**□**/■) and hydrosoluble fractions HF90/10 (**△**/**▲**). Data represent the mean ± SD of three independent experiments (each run in triplicate). The dotted vertical line corresponds to the maximum concentration of dry extract (MCE = 10,000 pg/µL) for matrix interferences.

For all positive fractions of *T. niloticus*, raw EC_50_ values expressed in pg/µL of dry extract were converted into µg flesh equiv./µL. For liposoluble fractions, the EC_50_ values were 46.86 ± 16.23 (*n* = 2), 0.23 ± 0.07 (*n* = 4) and 0.35 ± 0.07 (*n* = 4) µg flesh equiv./µL for LF70/30, LF90/10 and LF100, respectively, with L90/10 being the most potent of liposoluble fractions (Figure 3a). For hydrosoluble fractions, the EC_50_ estimated for HF90/10 and H100 were 20.36 ± 1.79 (*n* = 3) and 15.40 ± 4.34 (*n* = 3) µg flesh equiv./µL, respectively, with HF100 being the most potent of hydrosoluble fractions (Figure 3b). Conversely, HF50/50 was found nontoxic, while the activity detected in HF70/30 was likely due to the matrix effect (EC_50_ = 13,000 > MCE). Regarding *T. niloticus* collected in December 2014, typical P-CTXs’ responses were observed in only three fractions, i.e., LF90/10, LF100 and HF90/10 with corresponding EC_50_ values of 0.91 ± 0.06 (*n* = 3), 2.82 ± 0.29 (*n* = 3) and 23.04 ± 2.80 (*n* = 3) µg flesh equiv./µL, respectively (Figure 4a). As for *T. niloticus* collected in November 2016, only fraction LF90/10 displayed a response typical of CTXs activity, with an EC_50_ value of 2.29 ± 0.22 (*n* = 3) µg flesh equiv./µL (Figure 4b). For all *T. niloticus* samples, LF90/10 was the most potent fraction (Figure 4).

The toxin content in fractions positive in CBA-N2a was further estimated (Table 4). Regardless of the sampling date, ≈97% of the total toxin content was found in the liposoluble fractions, primarily in LF90/10 followed by LF100, vs. only 3% in the hydrosoluble fractions (Table 4). Additionally, while CTXs were detected in almost all fractions collected in July 2014, only three and one fractions were found to contain residual ciguatoxicity six months (December 2014) and 28 months (November 2016) after the report of the poisoning incident, respectively.

The total toxin contents in *T. niloticus* samples collected in Anaho Bay were estimated at 11.47 ± 3.91, 2.16 ± 0.17 and 0.67 ± 0.06 ng P-CTX-3C equiv./g of flesh in July 2014, December 2014 and November 2016, respectively. The total toxin content showed a five-fold decrease between July and December 2014 and a 4.5-fold decrease between December 2014 and November 2016. Overall, the toxicity in *T. niloticus* of Anaho Bay decreased 25-fold from July 2014 until November 2016.

The conversion of the CBA-N2a results in P-CTX-1B equivalents gave the following values of 15.45 ± 5.26, 2.91 ± 0.23 and 0.90 ± 0.08 ng P-CTX-1B equiv./g of flesh respectively, for July 2014, December 2014 and November 2016, which largely exceed the advisory levels of 0.01 ng P-CTX-1B equiv./g of flesh recommended by both the US-FDA and the EFSA [24,25]. These values expressed in P-CTX-1B equivalents are slightly higher than the values expressed in P-CTX-3C equivalents as the EC_50_ of these two toxins were 1.44 ± 0.70 and =1.94 ± 0.41 fg/µL, respectively, for P-CTX-3C and P-CTX-1B, in our CBA-N2a experiments.

2. Samples from Taiohae and Taipivai Bays

All liposoluble and hydrosoluble fractions obtained from *T. niloticus* collected from Taiohae (December 2014 and November 2016) and Taipivai (December 2014) were negative in OV^−^ and OV^+^ conditions (data not shown). These findings confirm that the ciguatoxicity observed in *T. niloticus* sampled from Anaho Bay is not the result of matrix interferences. 

**Figure 5:**
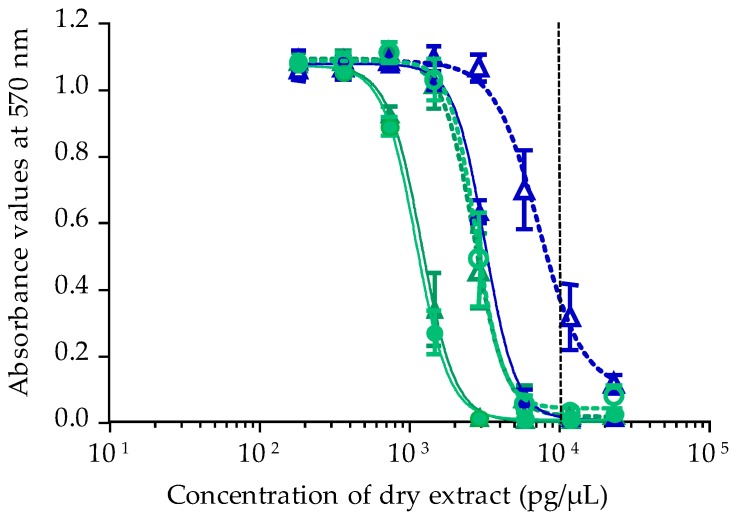
Dose-response curves of Neuro-2a cells in OV^−^ (open symbols) and OV^+^ (solid symbols) conditions, when exposed to increasing concentrations of *T. niloticus* fractions originated from Taipivai (November 2016) LF70/30 (**○**/●), LF90/10 (**△**/**▲**) and HF90/10 (**△**/**▲**) following the CBA-N2a procedure described in Section 4.4. Data represent the mean ± SD of two independent experiments (each run in triplicate). The dotted vertical line corresponds to the maximum concentration of dry extract (MCE = 10,000 pg/µL) for matrix interferences.

In contrast, *T. niloticus* collected from Taipivai in November 2016 displayed sigmoid dose response curves in both OV^−^ and OV^+^ conditions for three fractions, atypical of CTXs activity (Figure 5). 

For the three positive fractions, the corresponding EC_50_ values in OV^−^/OV^+^ conditions were: i.e., LF70/30: 20.49 ± 0.57 and 8.57 ± 0.09 µg/µL, respectively; LF90/10: 4.96 ± 0.15 and 2.22 ± 0.19 µg/µL, respectively; and HF90/10: ND and 78.23 ± 3.43 µg/µL, respectively. For all three fractions, EC_50_ values in OV^+^ conditions were higher than those obtained in OV^−^ conditions. LF70/30 and LF90/10 fractions had the same potency and were more potent than HF90/10. As these results were not typical of the P-CTX mode of action, no toxin content has been estimated.

### 2.3. Toxin Profiles in Tectus niloticus Toxic Fractions Using LC-MS/MS

#### 2.3.1. Detection of Pacific Ciguatoxins in *T. niloticus* from Nuku Hiva

**Figure 6:**
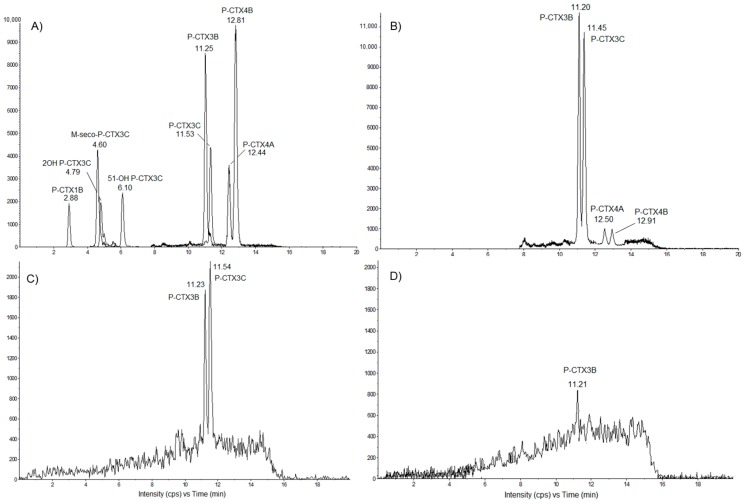
LC-MS/MS chromatograms obtained for (**A**) a mix of P-CTX standards (provided by Institut Louis Malardé) and Solid Phase Extraction-purified extracts of *Tectus niloticus* collected from Anaho Bay in (**B**) July 2014, (**C**) December 2014 and (**D**) November 2016. Chromatograms were acquired following the procedure described in Section 4.5, in positive multi-reaction monitoring (MRM) mode.

LC-MS/MS analyses confirmed the presence of P-CTXs in *T. niloticus* collected from Anaho Bay in July 2014, December 2014 and November 2016 (Figure 6A–D). 

In *T. niloticus* collected in July 2014 (Figure 6B), P-CTX-3B, P-CTX-3C, P-CTX-4A and P-CTX-4B were formally identified in comparison with standards (Figure 6A). For *T. niloticus* sampled in December 2014, P-CTX-3B and P-CTX-3C were formally identified in comparison with standards, whereas P-CTX-4A and P-CTX-4B were not detected (Figure 6C). As for *T. niloticus* collected in November 2016 (Figure 6D), only P-CTX-3B was formally identified in comparison with standards, whereas P-CTX-3C, P-CTX-4A and P-CTX-4B were not detected (Figure 6D).

Conversely, no CTXs were detected in *T. niloticus* from Taipivai Bay collected during the same periods.

The toxin profile of the *T. niloticus* extracts was determined and quantified using a calibration range of P-CTX-3C (Wako supplier) (Table 5). In the first extract (FE) obtained from *T. niloticus* collected in July 2014, P-CTX-3B was the most prevalent toxin congener (65%) with a toxin content twice as much as for P-CTX-3C (35%), while the more purified Solid Phase Extraction (SPE Si) extract displayed an even more diverse toxin profile including P-CTX-3B (65%), P-CTX-3C (28%) and P-CTX-4A/B (4% each), without significant loss (>90%).

The toxin profile assessed for *T. niloticus* extracts collected in December 2014 consisted of P-CTX-3C as the most prevalent toxin (61%), followed by P-CTX-3B (38%), whereas in the November 2016 samples, only P-CTX-3B accounted for the residual toxicity monitored in *T. niloticus*.

Based on the data presented in Table 5, the total toxin contents in *T. niloticus* samples were estimated at 23/21, 5.8 and 1.16 ng P-CTX-3C equiv./g of flesh in July 2014, December 2014 and November 2016, respectively (Table 5). These values indicate a 3.7-fold decrease in toxicity between July and December 2014, a 5-fold decrease between December 2014 and November 2016 or an overall 19-fold decrease from July 2014 until November 2016.

When converted into P-CTX-1B equivalents, the following toxin contents were obtained: 2.3/2.1, 0.58 and 0.12 ng P-CTX-1B equiv./g of flesh in July 2014, December 2014 and November 2016 samples, respectively, which again largely exceed the recommended action value of 0.01 ng P-CTX-1B equiv./g of flesh proposed by both the U.S. FDA and EFSA [24,25]. 

Despite the fact that toxin contents estimated from CBA-N2a and LC-MS/MS analysis appeared different by a factor of almost two, our findings indicate that: (i) the general trend observed in the decrease of *T. niloticus* toxicity over time from Anaho Bay remains consistent between these two analytical methods; and (ii) *T. niloticus* were able to accumulate CTXs in their tissues at levels well above the threshold indicated for human poisoning.

#### 2.3.2. Screening of *T. niloticus* Samples for Other Marine Toxins

As CBA-N2a results of *T. niloticus* samples from Taipivai in November 2016 were not characteristic of the presence of P-CTXs, a multi-toxin screening was conducted on *T. niloticus* samples from both Anaho (July 2014, December 2014 and November 2016) and Taipivai (November 2016). 

No NSP toxins (brevetoxin group), DSP toxins as okadaic acid (OA), dinophysistoxins (DTXs), azaspiracids (AZAs) and yessotoxins (YTXs), no pectenotoxins (PTXs), as well as FATs comprising gymnodimines (GYMs), spirolides (SPXs) and pinnatoxins (PnTXs) were found in *T. niloticus* from Anaho Bay. 

**Figure 7:**
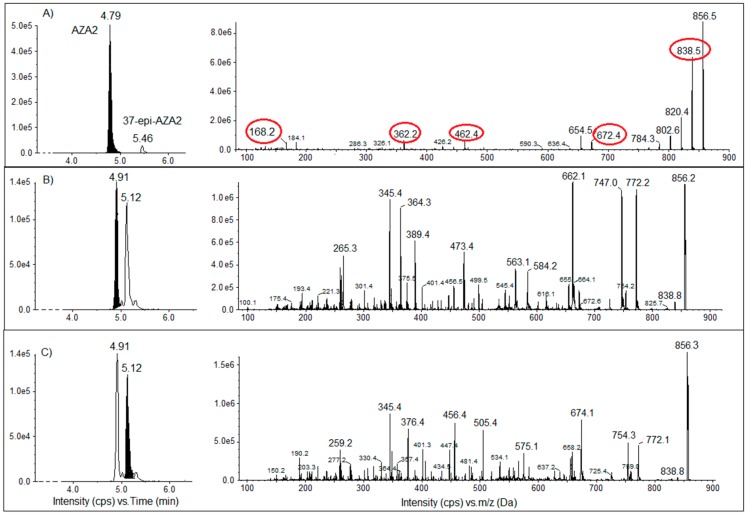
Chromatographic peaks of the *m*/*z* 856.6 ion with the corresponding MS/MS spectra of (**A**) AZA2 (AZA, azaspiracid) from National Research Council Canada (NRC) at 4.79 min; (**B**,**C**) *Tectus niloticus* extract from Taipivai collected in November 2016, at respectively, 4.91 min and 5.12 min. MS/MS spectra of the *m*/*z* 856.6 were obtained using positive enhanced product ion (EPI) mode with a collision energy (CE) of 50 eV and a collision energy spray (CES) of 20 eV. Specific fragment ions of the azaspiracid group are indicated in red.

Only *T. niloticus* samples from Taipivai in November 2016 presented two peaks with an *m*/*z* of the parent ion corresponding to that of AZA2, albeit with different retention times and spectral signatures (Figure 7).

Fragmentation of the parent ion *m*/*z* 856.6 was conducted using positive EPI mode on the API 4000Qtrap to verify if these intense peaks belong to the azaspiracid family. As previously described, azaspiracid analogs show characteristic fragmentation patterns in MS/MS analysis [26].

The MS/MS spectra obtained for the suspected peaks were compared to the fragmentation pattern of AZA2 (Figure 7), but the presence of known AZA analogs (AZA1–AZA32) could not be confirmed.

## 3. Discussion

A food poisoning outbreak involving nine tourists following the consumption of the gastropod *Tectus niloticus* was reported from Anaho Bay, Nuku Hiva Island (Marquesas archipelago, French Polynesia) in June 2014 [15,16]. All patients exhibited clinical symptoms typical of CFP [15]. As this poisoning incident involved an unusual vector of CFP, a toxicological survey of *T. niloticus* toxicity harvested from three distinct fishing sites in Nuku Hiva Island was conducted during a two-year period using both CBA-N2a and LC-MS/MS analysis.

CBA-N2a and LC-MS/MS results revealed the presence of high levels of P-CTXs only in *T. niloticus* specimens collected from Anaho Bay, in the same area where trochus specimens involved in the June 2014 mass-poisoning incident were harvested. Of note, the total toxin content in *T. niloticus* specimens on three distinct occasions, from July 2014 until November 2016, was found to be consistently above the safety limit recommended for human consumption, even 28 months after the poisoning outbreak occurred in Anaho Bay. Such findings suggest that *T. niloticus* is able to naturally bioaccumulate P-CTXs in its tissues and, hence, likely provides a potential bioaccumulation pathway for ciguatera toxins in marine food webs. Other marine gastropods in the *Tegulidae* and *Trochidae* families are known to bioaccumulate PSP toxins, such as *Tectus nilotica pyramida* and *Tectus pyramis* in Japan or *Tectus fenestratus* and *Trochus hanleyanus* endemic to the North-West Australian coast [27,28,29,30]. Indeed, trochus shells are found to graze on turf algae and biofilms growing on rocky and coral substrates [6,7]. Both field and laboratory feeding studies have established that *T. niloticus* feed on macroalgae belonging to Chlorophyta (Ulvaceae, Caulerpaceae) and Rhodophyta (Gracilariaceae, Solieriaceae), as well as diatoms (Bacillariaceae, Chaetocerotaceae, Naviculaceae), and other microalgae (Isochrysidaceae, Chlorodendraceae, Eustigmatophyceae) or suspended material mixed with sand and detritus [6]. These observations strongly suggest that *T. niloticus* feeding on macroalgae colonized by toxic *Gambierdiscus* cells are likely to concentrate algal ciguatoxins. 

In the present study, low densities of *Gambierdiscus* were consistently observed on macroalgal samples collected from the three study sites in November 2016. However, a previous field-survey conducted in Nuku Hiva Island showed that several species of macroalgae such as *Amphiroa fragilissima*, *Chlorodesmis fastigiata*, *Halimeda distorta* and *Chnoospora minima* can harbor high densities of *Gambierdiscus* cells, reaching 20,000 cells/g algae, most notably in Anaho Bay [31]. Concurrently, cell densities monitored on window screens deployed in Anaho, Taipivai and Taiohae Bays, by means of qPCR techniques, confirmed that *Gambierdiscus* populations are present in these three sites of Nuku Hiva Island. Estimation of the relative species distribution among Anaho Bay *Gambierdiscus* communities indicated that *G. polynesiensis* was the predominant species in this toxic area. *G. polynesiensis* is known as one of the most toxic species of *Gambierdiscus* described to date [32]. The toxin profile of two Polynesian strains of this species has been well documented by Chinain et al., 2010 [17], and includes P-CTX-3C, -3B, -4A, -4B and M-seco-CTX-3C as the major CTXs congeners produced by *G. polynesiensis* in culture. Since LC-MS/MS analyses showed that at least four of these P-CTXs analogs were present in *T. niloticus* toxic samples, it can be concluded that *G. polynesiensis* is a likely source of the CTX analogs detected in *T. niloticus* from Anaho Bay. Interestingly, a recent field survey conducted in Anaho Bay by means of solid phase adsorption toxin tracking (SPATT) technology also confirmed the presence of P-CTX-3B and -3C in the environment [33]. Taken together, all these observations confirm the high ciguatera risk status that characterizes Anaho Bay, as also evidenced by the epidemiological data available for Nuku Hiva Island (incidence rates of CFP varying from 37–101/10,000 inhabitants between 2012 and 2016, www.ciguatera-online.com).

Conversely, in Taiohae and Taipivai Bays, the dominant species in the benthic communities recovered from window screens was *G. carpenteri*, while *G. pacificus* was detected only at trace concentrations in WS samples in all sites. Contrary to *G. polynesiensis*, *G. carpenteri* and *G. pacificus* are not known to produce significant amounts of CTX nor maitotoxins (MTXs) [17,32,34,35,36,37], consistent with the fact that no P-CTXs were detected in *T. niloticus* samples collected in November 2016 from these two study sites. However, the presence of a highly toxic strain of *G. polynesiensis* TIO-10 among the clonal cultures established in the laboratory from cell isolates from Taiohae Bay suggests that potential health hazards may be associated with the consumption of trochus harvested in this area. This emphasizes the need to keep this site under surveillance, along with the Anaho study site. 

There are several possible explanations for the occurrence of the toxic algae in Anaho Bay. CFP events are triggered by both environmental and biological factors. Chinain et al., 1999 [38], have shown the existence of a seasonal trend in *Gambierdiscus* proliferation in French Polynesia, with maximum densities occurring during the hot season. CFP risk is also significantly increased in highly degraded coral reef ecosystems [18,20,39], which was the case in Anaho Bay (Chinain, personal communication). While anthropogenic discharges are unlikely in this uninhabited and difficult to access area, the high hydrodynamic conditions that prevail in this area throughout the hot season may partly explain the degraded status of this site. 

According to Litaker et al., 2010 [40], CFP events are driven more by inherent differences in species toxicity than by environmental modulation. Chinain et al., 1999 [38], have also speculated that the toxicity in a given area is mainly dependent on the clonal nature of cells that coexist within local *Gambierdiscus* populations. In other words, the high proportion of *G. polynesiensis* cells in Anaho Bay may account for the high CFP risk in this area. Recent studies have shown that temperature, salinity and irradiance tolerance may vary greatly across multiple species of *Gambierdiscus* [41,42,43,44]. Such differences may in turn influence the growth and distribution of these species in a given area. Additionally, since *Gambierdiscus* is often found as part of a mixed assemblage of benthic dinoflagellates on macroalgal substrates, the existence of allelochemical-mediated growth stimulation or inhibition mechanisms may help explain why *G. polynesiensis* turned out to be the dominant species in Anaho Bay [44,45,46].

Based on LC-MS/MS analyses, four distinct P-CTX analogs were currently detected in *T. niloticus* tissues, namely P-CTX-3B, -3C, -4A and -4B, with P-CTX-3B as the major congener. This result is consistent with previous findings indicating that P-CTX-3B is also the predominant congener detected in giant clams (*Tridacna maxima*) experimentally fed *G. polynesiensis* cells [23]. 

In the present study, the toxicity in *T. niloticus* samples from Anaho Bay was monitored at different time periods corresponding to 1, 6 and 28 months after the mass-poisoning was reported. The total toxin content estimated in toxic fractions based on CBA-N2a and LC-MS/MS data showed a 19–25-fold decrease in the overall ciguatoxicity of these samples (depending on the detection test) between July 2014 and November 2016, suggesting a slow depuration rate for CTXs in these marine invertebrates. However, this finding may also be confounded by additional accumulation over the two-year period, even through low levels of *Gambierdiscus*. Dynamics of PSP toxins accumulation and elimination is well documented in a variety of bivalve mollusks such as mussels, scallops, oysters and clams fed toxic dinoflagellates (*Alexandrium*, *Ostreopsis*, *Azadinium*) [47,48,49,50,51]. In contrast, the processes controlling the uptake, metabolization and depuration of CTXs are still poorly addressed in marine invertebrate species prone to ciguatera [23], especially gastropods, stressing the urgent need for further investigations in this field of research, since such knowledge will greatly benefit both ciguatera risk management programs and predictive models of CTX accumulation in these organisms. 

The implication of various marine invertebrates in ciguatera events is well documented in the Pacific region and, to a lesser extent, in Atlantic localities. In addition to poisoning cases involving *Tridacna maxima* (bivalve mollusk) or *Tripneustes gratilla* (echinoderms) frequently observed in French Polynesia, New Caledonia and Cook islands [19,21,22,52], toxic lobsters *Panulirus penicillatus* and octopus have also been reported in the Republic of Kiribati [53]. More recently, ciguatoxin congeners were also evidenced in the starfish *Ophidiaster ophidianus* and *Marthasterias glacialis* collected from the northwestern Moroccan coast [54]. In some instances where atypical ciguatera symptoms were observed (e.g., rapid onset of the clinical symptoms, unusual severity of the poisoning which necessitated the hospitalization of patients), the potential contribution of marine cyanobacteria belonging to the Oscillatoriales (e.g., *Trichodesmium*, *Oscillatoria* and *Hydrocoleum*) was suspected and led to the description of a new ecotoxicological phenomenon tentatively designated ciguatera shellfish poisoning (CSP) [19,52]. Further toxicological analysis revealed the production of a variety of toxic metabolites in these cyanobacteria, including ciguatoxin-like compounds, anatoxin-a and homoanatoxin-a, palytoxin and 42-hydroxy-palytoxin [19,52,55,56,57,58]. Because similar atypical symptoms were also observed in patients poisoned in Anaho Bay, a multi-toxin screening by means of LC-MS/MS targeted at paralytic shellfish poisoning toxins, diarrhetic shellfish poisoning toxins, cyclic imines (fast action toxins (FAT)) and brevetoxins is currently underway on *T. niloticus* samples collected in Nuku Hiva Island in November 2016. Preliminary results strongly suggest the presence of yet undescribed AZAs (azaspiracid toxins) analogs (the presence of two peaks with an *m*/*z* equivalent to the peak of AZA2, but with different retention times), at least in the trochus samples from Taipivai Bay. Analyses for the presence of *Azadinium* spp. cells in water samples collected from this site were negative.

*T. niloticus* is of high value for many island communities in PICTs where it represents not only a significant source of revenue, but also a valuable nutritional resource. This study is the first to provide evidence that the consumption of trochus shell meat may represent a potential health risk in French Polynesia. Indeed, high levels of CTXs exceeding the advisory levels recommended by the European Food Safety Authority (EFSA) [24] and the Food and Drug Administration (FDA) [25] were detected in the tissues of *T. niloticus* collected from Anaho Bay, a long-standing ciguatera hotspot in Nuku Hiva Island. Moreover, the potential presence of other toxic compounds in these marine invertebrates points to the importance of: (i) maintaining an eco-toxicological surveillance in areas already identified to present a high risk for poisoning; (ii) document the prevalence of such poisoning events in other localities of French Polynesia; and (iii) conduct sustained educational interventions to increase public awareness in order to minimize the risk of seafood intoxication by ingestion of *T. niloticus* in both local populations and visitors.

## 4. Materials and Methods

### 4.1. Study Sites

**Figure 8:**
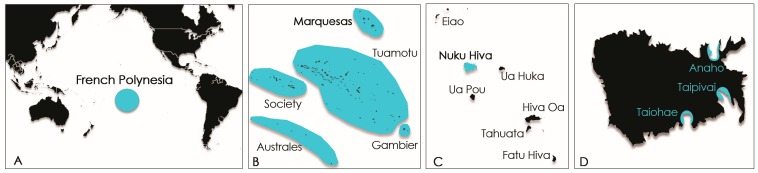
Maps of French Polynesia (**A**,**B**) and Nuku Hiva Island (Marquesas archipelago), (**C**) showing the locations of the 3 study sites and (**D**) areas sampled in this study.

Samples (macroalgal substrates, window screens and *T. niloticus* specimens) were collected from 3 distinct study sites on Nuku Hiva Island (Marquesas archipelago, French Polynesia), namely Anaho, Taipivai and Taiohae bays located in the northern, eastern and southern coasts of the Island, respectively (Figure 8). Anaho Bay has been regarded as a long-standing hotspot of ciguatera since 2004 [31].

### 4.2. Biological Material and Sampling Procedures 

#### 4.2.1. *Gambierdiscus* Samples and qPCR Assays

Wild samples of *Gambierdiscus* analyzed in this study from Anaho, Taipivai and Taiohae bays, using both the natural (i.e., macroalgae) and the artificial (i.e., window screens) substrate methods were collected in November 2016 [59]. Briefly, ≈200 g of turf-like and *Halimeda micronesia* macroalgal hosts were collected at water depths of 1–5 m and examined for the presence of *Gambierdiscus* cells. Macroalgal samples were sealed within plastic bags underwater and shaken and kneaded vigorously to dislodge dinoflagellate cells. The detrital suspension was then successively filtered through 125-, 40- and 20-µm mesh sieves and the 40- and 20-µm fractions preserved in 50 mL of 5% formalin-seawater. Cell densities were assessed microscopically from 100-µL aliquots of these two fractions. Values were expressed in cells/g algal wet weight and represented the mean number of cells enumerated on *n* (=2–8) sub-samples of the same host algae species [20]. The artificial substrate method used 150 cm^2^ window screen (WS) devices assembled and deployed in the same areas. A total of 6 WSs were deployed per areas; 100% of the WS were recovered. After 24 h, WS were harvested with 250 mL of sea water and shaken to dislodge the cells from the WS. The entire volume was filtered through 10-μm polycarbonate filters that were replaced as the filters became obstructed. Then, all filters used to process individual samples were transferred into 15 mL tubes with 8 mL of sterile filtered sea water. Before removing the filters, the tubes were shaken to dislodge *Gambierdiscus* cells. Enumeration of cells was performed microscopically on 1 mL aliquots and by qPCR estimation on the remaining 7 mL as described below. Cell concentrations were expressed as cells/150 cm^2^ [59].

Concurrently, 17 in vitro culture clones of *Gambierdiscus* spp. were also established in the laboratory from single cell isolates collected from WS samples, according to the method used by Chinain et al., 2010 [20]. 

Semi-quantitative, species-specific qPCR assays were used to survey the WS samples for relative cell abundance and *Gambierdiscus* species distribution. Each 7-mL WS aliquot was filtered onto 47-mm 8-µm polycarbonate filters, and DNA was extracted from each filter as described by Vandersea et al., 2012 [60], using the Power Soil DNA isolation Kit (Quiagen, Hilden, Germany) following the manufacturer’s protocol, except that 350 µL of cell lysate rather than the prescribed 450 µL were processed. The DNA extracts were eluted from the mini columns using 50 µL of elution buffer and stored at 4 °C. Taxonomic identification of in vitro cultures and enumeration of wild cells collected on WS were conducted using qPCR assays described by Vandersea et al., 2012 [60], using species-specific primers for *G. belizeanus*, *G. caribaeus*, *G. carpenteri*, *G. carolinianus*, *G. ruetzleri* and *Gambierdiscus* ribotype 2. The presence and abundance of Pacific *Gambierdiscus* species, i.e., *G. polynesiensis*, *G. toxicus*, *G. pacificus* and *G. australes*, were assessed using species-specific qPCR primer sets (Table 6) and qPCR conditions. 

PCR assays were performed using an Eppendorf Mastercycler^®^ ep RealPlex 4 system with white Eppendorf real-time tube strips (Eppendorf North America, Inc., Westbury, New York, NY, USA) and a total reaction volume of 10.5 µL per tube. Each PCR reaction mixture contained 4.5 µL of 5 Prime RealMasterMix SYBR ROX 2.5× (0.05 units/µL Taq DNA polymerase, 10 mM Mg(CH_3_COO)_2_, 1.0 mM dNTPs, 20X SYBR^®^ Green solution) each primer at a concentration of 0.15 µM, 4.7 µL of sterile deionized water and 1 µL of template DNA. Thermal cycling conditions included denaturation at 95 °C for 2 min followed by 40 cycles at 95 °C for 10 s, annealing for 15 s at 60 °C with a subsequent extension at 68 °C for 20 s. The fluorescence threshold was determined by the Eppendorf RealPlex 4 analytical software, and the PCR cycle during which fluorescence crossed the threshold was designated the quantification cycle (Cq). A melting curve analysis was performed following thermal cycling to check the specificity of the PCR reactions. The melting curve profile consisted of denaturation at 95 °C for 15 s followed by an annealing step for 15 s at 60 °C. The fluorescence was continuously monitored during a steady 20-min temperature ramp from 60–95 °C, which was held at 95 °C for 15 s. The melting curve analysis was conducted by comparing the melting temperature peak of positive control DNA to other experimental DNA samples. A limit of ±0.5 °C for melting temperature peak shift was set as the cutoff for species-specific amplifications.

To quantify *Gambierdiscus* cells collected using WS, the construction of qPCR amplicon standard curves was necessary. rDNA fragments for each species were PCR amplified using primers that flanked the qPCR assay target sites as described by Vandersea et al., 2012 [60]. Cell-based standard curves for *G. polynesiensis*, *G. toxicus*, *G. pacificus*, *G. australes*, *G. caribaeus* and *G. carpenteri* were constructed as previously described. To obtain qPCR cell number estimates, the ratio of extractable PCR amplicons per cell was determined. The number of extractable PCR amplicons per cell was calculated by solving the regression equations derived from the diluted PCR amplicon-based standard curves using Cq values acquired from qPCR amplification of known numbers of *Gambierdiscus* cells. The results of this procedure enabled species-specific quantitative cell number estimates in each WS sample that was processed.

For identification at the species level, qPCR assays using the same species-specific primer sets previously described were also conducted on the 17 *Gambierdiscus* clonal cultures established from Nuku Hiva Island.

#### 4.2.2. *Tectus niloticus* Samples

Since *T. niloticus* is currently listed among protected marine species in French Polynesia and is thus subject to a permanent fishing ban, sampling in the frame of this study was made possible through an authorization issued by the Marine and Mining Resources Directorate (Direction des Resources Marines et Minières, DRMM) of French Polynesia. Trochus specimens were collected from Anaho Bay (Figure 8) by staff members of the Public Health Directorate of Nuku Hiva Island in July 2014 and December 2014, i.e., 1 month and 6 months, respectively, after the first report of the poisoning outbreak in Anaho. A third sampling campaign conducted by the laboratory took place in Anaho, Taipivai and Taiohae Bays (Figure 8) in November 2016, i.e., 28 months after the poisoning incident occurred. The basal diameter of the shell was measured individually for all trochus samples (Table 7), and shells were sent to the DRMM or destroyed, as it is forbidden to keep them. For samples sent by the Public Health Directorate of Nuku Hiva Island in July 2014 and December 2014, the trochus specimens were boiled in order to get the meat out. The whole flesh was weighed individually and pooled before chemical extraction (Table 7). For samples collected in November 2016, the whole fresh flesh was removed by breaking the shell, weighed individually, pooled and frozen for transport to the laboratory. Then, samples were lyophilized before chemical extraction (Table 7).

### 4.3. Extraction Procedures

In Pacific Island Countries and Territories like French Polynesia, *T. niloticus* meat is highly esteemed, and the entire trochus is consumed with or without the viscera depending on the traditional practices of the consumers. Consequently, the whole animal was extracted following a protocol adapted from Roué et al., 2016 [23]. Briefly, each pooled *T. niloticus* sample was extracted twice in methanol (MeOH) and twice in 50% aqueous MeOH, under sonication for 4 h. After one night at −20 °C, the crude extracts were centrifuged, and the supernatants were pooled and dried under vacuum. The resulting crude extract was further partitioned between dichloromethane (CH_2_Cl_2_) and 60% aqueous MeOH (=hydrosoluble fraction (HF)). The resulting CH_2_Cl_2_ phase (liposoluble fraction (LF)) was dried under vacuum and further defatted by a second solvent partition using cyclohexane and 80% aqueous MeOH (=LF). The 60% aqueous MeOH phase (HF) and 80% aqueous MeOH phase (LF) were then evaporated and further purified on C_18_ Sep-Pak cartridges (Waters^®^, Saint-Quentin, France). For HF, the columns were pre-conditioned with 30% aqueous MeOH before loading extracts, washed with 30% aqueous methanol and then eluted successively with 50%, 70% and 90% aqueous methanol and pure methanol, resulting in 4 distinct hydrosoluble fractions, i.e., HF50/50, HF70/30, HF90/10 and HF100, respectively. For LF, the columns were pre-conditioned with 70% aqueous MeOH before loading extracts, washed with 70% aqueous methanol and eluted successively with 90% aqueous methanol and pure methanol, leading to 3 distinct liposoluble fractions, i.e., LF70/30, LF90/10 and LF100. All these fractions were then dried in a SpeedVac concentrator, weighted and stored at +4 °C until tested for their toxicity. 

For *Gambierdiscus* spp. culture samples, only the dichloromethane phase in which lipid-soluble toxins such as CTXs are recovered was kept, dried and stored until tested for its toxicity, as described by Chinain et al., 2010 [20].

### 4.4. Cell-Based Assay Using Neuroblastoma Cells

*Gambierdiscus* spp. and *T. niloticus* extracts were analyzed for their toxicity using the neuroblastoma cell-based assay (CBA-N2a), a test designed to detect the presence of toxins acting on voltage-gated sodium channels (VGSCs) such as brevetoxins and CTXs, which are both VGSCs activators [61].

The procedure for CBA-N2a follows the method previously described by Roué et al., 2016 [23]. Briefly, a density of 45,000 neuroblastoma (neuro-2a) cells/200 µL/well in 5% fetal bovine serum RPMI-1640 supplemented medium was seeded in a 96-well microtiter plate. After 20–24 h of growth at 37 °C, in a humidified 5% CO_2_ atmosphere, all wells reached 100% confluence. Then, the medium was replaced by 200 µL of fetal bovine serum (FBS) 2.5% RPMI-1640 for half of the wells and 200 µL of the same medium containing an ouabain-veratridine solution (OV) at a concentration of 100/10 µM for the other half of the wells. 

Untreated cells without ouabain and veratridine added (OV^−^ conditions) or treated cells with ouabain and veratridine mixture (OV^+^ conditions) were first exposed to 4 P-CTXs standards: using a serial dilution 1:2 of eight concentrations ranging from 0.74–95.24 fg/µL for P-CTX-4A, P-CTX-4B, and P-CTX-3B and 0.15–19.05 fg/µL for P-CTX-3C. 

The maximum concentration of dry extract (MCE) that does not induce unspecific mortalities in neuro-2a cells was established at 10,000 pg/µL for both *Gambierdiscus* and trochus matrices. All *T. niloticus* fractions were tested in CBA-N2a using a serial dilution 1:2 of eight concentrations ranging from 37–9524 pg/µL of dry extract for most of the fractions, except for LF90 and LF100, which were tested at concentrations ranging from 12–1587 pg/µL of dry extract. For *Gambierdiscus* spp. cultures, extracts were first tested at a single concentration of 9524 pg/µL, and if toxic, a full dose-response curve was generated by testing eight distinct concentrations ranging from 0.15–19.05 pg/µL of dry extract. Each concentration was tested in OV^−^ and OV^+^ conditions, in triplicate per plate, in two to four independent experiments.

Following another 20–22-h incubation period, cell viability was assessed using the MTT assay. The incubation medium was removed, and 60 µL of RPMI-1640 medium containing 0.8 mg/mL of 3-(4,5-dimethylthiazol-2-yl)-2,5-diphenyl tetrazolium bromide (MTT) were added to each well. The plates were incubated for 45 min at 37 °C. Finally, the MTT was discarded, and 100 µL of dimethyl sulfoxide (DMSO) were added to each well to dissolve the formazan. 

The absorbance was measured at 570 nm using a plate reader (iMark Microplate Absorbance Reader, BioRad, Marnes la Coquette, France). For all experiments, absorbance values of OV^−^ and OV^+^ control wells were around 1 corresponding to 100% viability. Absorbance data were fitted to a sigmoidal dose-response curve (variable slope) based on the four-parameter logistic model (4PL) allowing the calculation of EC_50_ values using Prism v6.0.7 software (GraphPad, San Diego, CA, USA). Since raw results for all extracts were obtained in pg/µL of dry extract, the EC_50_ values for *T. niloticus* and *Gambierdiscus* samples were further expressed in µg flesh equiv./µL and in cells equiv./µL, respectively.

The toxin content (T) in the extracts was estimated using the following formula T = (P-CTX-3C EC_50_/sample EC_50_) and was expressed in ng P-CTX-3C equiv./g wet weight of flesh for *T. niloticus* and in pg P-CTX-3C equiv./cell for *Gambierdiscus* samples. The limit of detection (LOD) was estimated according to the method of Caillaud et al., 2012 [62], and Roué et al., 2016 [23], and was 0.17 fg P-CTX-3C equiv./cell and 0.02 ng P-CTX-3C equiv./g wet weight of flesh for *Gambierdiscus* and *T. niloticus*, respectively. 

### 4.5. Liquid Chromatography Coupled with Tandem Mass Spectrometry 

Analyses using liquid chromatography coupled with tandem mass spectrometry (LC-MS/MS) were conducted on freeze-dried samples of *T. niloticus* samples collected in July and December 2014 and November 2016.

Samples were extracted as follows: a homogenate of freeze-dried meat was extracted twice with acetone. After centrifugation, the supernatants were pooled and evaporated by rotary evaporation. The dry extract was dissolved in aqueous methanol (90:10), and a liquid-liquid partition with hexane was carried out. The 90% methanolic fraction was evaporated and then dissolved in 100% methanol. An aliquot of this extract (first extract, FE) was filtered over 0.2 μm before being analyzed by LC-MS/MS (Sciex, Kenwood, CA, USA).

The FE extract was then purified on silica cartridges Solid Phase Extraction (SPE Si) type Florosil^®^ (Waters, Saint-Quentin, France), and the resulting fraction (SPE Si extract) evaporated under nitrogen and resuspended in 100% methanol prior to LC-MS/MS analysis.

The resulting fractions or extracts were analyzed by LC-MS/MS in MRM (multi-reaction monitoring) mode on a triple quadrupole API4000 QTrap (Sciex, Redwood, CA, USA). Biological samples and P-CTXs were analyzed using an LC-MS/MS method adapted from Yogi et al., 2011 [63]. The instrument used was an LC system (UFLC XR Nexera, Shimadzu, Kyoto, Japan) coupled to a hybrid triple quadrupole-linear ion trap mass spectrometer (API-4000Qtrap, Sciex, Kenwood, CA, USA) equipped with a turboV^®^ ion spray interface. A 1.8-µm C_18_ Zorbax Eclipse plus column (50 × 2.1 mm, Agilent technologies, Santa Clara, CA, USA) was employed at 40 °C and eluted at 400 µL/min with a linear gradient. Eluent A is water, and Eluent B is methanol, both eluents containing 2 mM ammonium formiate and 50 mM formic acid. The elution gradient ran from 78–88% over 10 min and was held for 4 min before re-equilibration during 5 min.

Mass spectrometry detection was operated in positive mode and using multiple reaction monitoring (MRM) (Analyst software, Sciex, Kenwood, CA, USA). The pseudomolecular ions [M + NH_4_]^+^ and [M + H]^+^ were selected as precursor ions. The ions resulting in the successive losses of NH_4_ and/or water molecules were selected as product ions (Table 8). The MRM experiments were established by using the following source settings: curtain gas set at 25, ion spray at 5500 V, a turbogas temperature of 300 °C, Gas 1 set at 40 and Gas 2 set at 60 psi with an entrance potential of 10 V and declustering potential of 105 V.

Data processing and analysis were carried out using Analyst software (Sciex, Kenwood, CA, USA). Quantification was performed by linear calibration using P-CTX-3C standard (Wako chemicals GmbH, Neuss, Germany). To complete chromatogram profiles, a mix of standards (P-CTX-1B, P-CTX-3B, P-CTX-4A, P-CTX-4B, M-*seco* P-CTX-3C and 51-OH P-CTX-3C) provided by Louis Malardé Institute (ILM, Tahiti, French Polynesia) was injected in the sequence.

In addition to the detection of P-CTXs, other marine biotoxins were investigated according to methods previously described [64,65,66]: neurologic shellfish toxins (NSP), i.e., brevetoxins (PbTX1–PbTX10), paralytic shellfish poisoning (PSP) toxins, i.e., carbamates (STX, NEO-STX, GTX1–GTX4), N-sulfocarbamoyl (GTX5, GTX6 and C1–C4) and decarbamoyls (dcSTX, dcNEO, dcGTX1–dcGTX4); diarrhetic shellfish poisoning (DSP) toxins, i.e., okadaic acid (OA), dinophysistoxins (DTXs), pectenotoxins (PTXs), azaspiracids (AZAs) and yessotoxins (YTXs); cyclic imines (fast action toxins (FAT)): gymnodimines (GYMs), spirolides (SPXs) and pinnatoxins (PnTXs). 

## Figures and Tables

**Table 1:** Densities ^1^ of *Gambierdiscus* spp. found in macroalgal substrates in 3 distinct locations of Nuku Hiva Island.

Site	(*n*)	Macroalgae	
		Genus	Total ^1^
Anaho	8	Turf, *Halimeda*	1.5 ± 2.6
Taipivai	2	*Halimeda micronesica*	0
Taiohae	-	NF ^2^	-

^1^ Densities counted manually and expressed in cells/g algal wet weight; ^2^ NF = not found.

**Table 2:** Semi-quantitative qPCR estimates of *Gambierdiscus* species composition identified from window screen sampling ^1^ in Nuku Hiva Island.

Species	Location		
	Anaho Bay *n* = 6	Taipivai *n* = 6	Taiohae *n* = 6
*G. caribaeus*	<1%	<1%	<1%
*G. carpenteri*	17%	90%	88%
*G. pacificus*	<1%	<1%	<1%
*G. polynesiensis*	82%	10%	10%
*G. toxicus*	<1%	<1%	1%
Total cells	~2900	~415	~420

^1^ 150 cm^2^ window screen.

**Table 3:** Identification to the species level of the in vitro culture strains of *Gambierdiscus* spp. isolated from window-screen samples deployed in Nuku Hiva Island.

Site	*Gambierdiscus* Species			
	*n* ^1^	*G. polynesiensis*	*G. carpenteri*	*G. pacificus*
Anaho	11	0	11	0
Taipivai	2	0	1	1
Taiohae	4	1	1	2

^1^ *n* = number of cultured strains established in the laboratory.

**Table 4:** Toxin content estimates in *T. niloticus* samples collected from Anaho Bay in July 2014, December 2014 and November 2016. Toxicity data were obtained using CBA-N2a. Each value represents the mean ± SD (*n* = 2–4 experiments).

Fractions	Toxin Content ^1^		
	July 2014	December 2014	November 2016
LF70/30	0.03 ± 0.01	ND ^2^	ND
LF90/10	6.63 ± 1.95	1.59 ± 0.11	0.67 ± 0.06
LF100	4.63 ± 1.63	0.51 ± 0.05	ND
HF50/50	ND	ND	ND
HF70/30	ND	ND	ND
HF90/10	0.07 ± 0.01	0.06 ± 0.01	ND
HF100	0.10 ± 0.03	ND	ND
Total toxin content	11.47 ± 3.91	2.16 ± 0.17	0.67 ± 0.06

^1^ Results expressed in ng P-CTX-3C equiv./g of flesh. ^2^ ND: not detectable.

**Table 5:** Estimation of the relative concentrations of P-CTXs congeners in *T. niloticus* collected in Anaho Bay using LC-MS/MS.

Date	Fractions	P-CTX-3C	P-CTX-3B	P-CTX-4A	P-CTX-4B	Total
July 2014	First Extract FE	8.1	14.81	<LD ^1^	<LD	23
	SPE Si Extract	5.8	13.0	0.9	0.8	21
December 2014	SPE Si Extract	3.57	2.2	<LD	<LD	5.8
November 2016	SPE Si Extract	<LD	1.16	<LD	<LD	1.16

^1^ Results expressed in ng P-CTX-3C equiv./g of flesh with a detection limit of 0.05 ng P-CTX-3C equiv./g of flesh.

**Table 6:** *Gambierdiscus* species-specific qPCR primer sets used in this study.

Species	Name of Primers	Sequences of qPCR Primers
*G. australes*	GaustF10	5′-ATTGCTGTGTGAATACAGGTAA-3′
	GaustR10	5′-CAAGCACTGCCCACAGATAC-3′
*G. pacificus*	GpacifITSF1	5′-AATTCGAAACAGATGTGCATGG-3′
	GpacifITSR1	5′-GCCAAAGACAGCACTGATGAC-3′
*G. polynesiensis*	PolyITSF1	5′-TGTGTGCACGTGTGTGTATGG-3′
	PolyITSR1	5′-CGCACCACCGGCGCACAG-3′
*G. toxicus*	GtoxITSF1	5′-TGAGACAGACGTGCATGGTTG-3′
	GtoxITSR1	5′-CCAACAGCAGCACTGATGAAT-3′
*G. belizeanus*	GbelizeF1	5′-TAGAGGAATTGACACAAACTTG-3′
	GbelizeR1	5′-CATCAGGGTTTTCAGGTCAAA-3′
*G. caribaeus*	GcaribF3	5′-TGTCTTTGACTGGATGACTGT-3′
	GcaribR8	5′-TGTCTCCAACATGCTGGCAC-3′
*G. carpenteri*	GcarpenteriF1	5′-GGTGCTGTTGTGTGACCATA-3′
	GcarpenteriR3	5′-CTGGCAGTGGAAGCTGACA-3′
*G. carolinianus*	GcarolinF2	5′-TAAATGAGAAAGGACGCAGC-3′
	GcarolinR5	5′-CACCTCTCACTTCAAATTGG-3′
*F. ruetzleri*	GrutzITSF3	5′-TGGATAACACCATGGGAAGTC-3′
	GrutzITSR4	5′-TTCCCAGCTTCGAGGGGAAA-3′
*Gambierdiscus* ribotype 2	RiboII-F1	5′-TTGGAGAGTGAATCTTGTCTT-3′
	RiboII-R1	5′-CGGTATCTGGCTTTGCGTG-3′

**Table 7:** Morphological features of *Tectus niloticus* samples.

Island	Area	Date	*n*	Shell Size ^1^ Basal Diameter (mm)	Whole Flesh Weight (g)
Nuku Hiva	Anaho	July 2014	13	117.7 ± 7.0	57.63 ± 15.2 ^2^
		December 2014	10	65.0 ± 8.8	38.2 ± 10.4 ^2^
		November 2016	12	117.0 ± 5.0	87.8 ± 19.4 ^3^
	Taipivai	December 2014	13	62.3 ± 7.6	36.1 ± 8.8 ^2^
		November 2016	10	121.0 ± 4.6	100.1 ± 7.4 ^3^
	Taiohae	November 2016	12	115.0 ± 8.0	85.0 ± 16.9 ^3^

^1^ Shell size (basal diameter in mm). ^2^ Whole flesh weight boiled (g) and ^3^ whole fresh flesh weight (g).

**Table 8:** Mass spectrometer parameters.

Compound	Precursor Ion (Q1) *m*/*z*	Product Ion (Q2) *m*/*z*	Collision Energy (CE, eV)	Collision Exit Potential (CXP, eV)	Retention Time (RT, min)
P-CTX1B	1128.6	1093.6	20	12	2.9
		1075.6	30	12	
P-CTX3C and P-CTX3B	1040.6	1005.6	30	12	11.3 and 11.5
	1023.6	1005.6	20	12	
P-CTX4A and P-CTX4B	1078.6	1043.6	30	12	12.4 and 12.8
	1061.6	1043.6	20	12	
2,3-diOH-P-CTX3C	1074.6	1057.6	30	12	
	1057.6	1039.6	20	12	
51-OH-P-CTX3C	1056.6	1021.6	30	12	6.1
	1039.6	1021.6	20	12	
M-*seco*-P-CTX3C	1041.6	1023.6	30	12	4.6
		1005.6	20	12	
P-CTX2 and P-CTX3	1112.6	1077.6	20	12	
		1059.6	30	12	
2-OH-P-CTX3C and 3-OH-P-CTX3C	1058.6	1023.6	30	12	4.8
		1005.6	20	12	
